# An Evolved 5′ Untranslated Region of Alfalfa Mosaic Virus Allows the RNA Transport of Movement-Defective Variants

**DOI:** 10.1128/jvi.00988-22

**Published:** 2022-10-31

**Authors:** David Villar-Álvarez, Vicente Pallás, Santiago F. Elena, Jesús A. Sánchez-Navarro

**Affiliations:** a Instituto de Biología Molecular y Celular de Plantas, CSIC-Universitat Politècnica de València, Valencia, Spain; b Instituto de Biología Integrativa de Sistemas, CSIC-Universitat de València, Paterna, Valencia, Spain; c Santa Fe Institute, Santa Fe, New Mexico, USA; Emory University School of Medicine

**Keywords:** cell-to-cell and systemic movement, virus particles, AMV model system, BMV

## Abstract

Although the coat protein (CP) has a relevant role in the long-distance movement of alfalfa mosaic virus (AMV) and brome mosaic virus (BMV), its precise function is not fully understood. Previous results showed that a specific interaction between the C termini of the movement protein (MP) and the cognate CP is required for systemic transport. Thus, we have performed a compensatory evolution experiment using an AMV RNA3 derivative defective in long-distance transport that carries a BMV MP lacking the C-terminal 48 residues and unable to interact with the AMV CP. After several passages, five independent evolution lineages were able to move long distance. The analysis of the viral RNA of these lineages showed the presence of three different modifications located exclusively at the 5′ untranslated region (5′ UTR). The three evolved 5′ UTR variants accumulated comparable levels of viral RNA and CP but reduced the accumulation of virus particles and the affinity between the 5′ UTR and the AMV CP. In addition, the evolved 5′ UTR increased cell-to-cell transport for both the AMV RNA3 carrying the BMV MP and that carrying the AMV MP. Finally, the evolved 5′ UTRs allowed the systemic transport of an AMV RNA3 carrying a CP mutant defective in virus particles and increased the systemic transport of several AMV RNA3 derivatives carrying different viral MPs associated with the 30K superfamily. Altogether, our findings indicate that virus particles are not required for the systemic transport of AMV but also that BMV MP is competent for the short- and long-distance transport without the interaction with the CP.

**IMPORTANCE** The results obtained in the present work could challenge the view of the role of the virus particle in the systemic transport of plant viruses. In this sense, we show that two different MPs are competent to systemically transport the AMV genome without the requirement of the virus particles, as reported for viruses lacking a CP (e.g., *Umbravirus*). The incapability of the viral MP to interact with the CP triggered virus variants that evolved to reduce the formation of virus particles, probably to increase the accessibility of the MP to the viral progeny. Our results point to the idea that virus particles would not be necessary for the viral systemic transport but would be necessary for vector virus transmission. This idea is reinforced by the observation that heterologous MPs also increased the systemic transport of the AMV constructs that have reduced encapsidation capabilities.

## INTRODUCTION

For the establishment of systemic infection, plant viruses need to replicate in an infected cell and colonize adjacent cells to eventually reach the vascular tissues (1, 2). Viruses move from one cell to another through cell wall connections known as plasmodesmata. To do this, viruses express movement proteins (MPs) capable of interacting with plasmodesmata and modifying their size exclusion limit, allowing their intercellular movement (3). Movement from the infected cell to adjacent healthy cells occurs either in the form of viral ribonucleoprotein (vRNP) complexes, in the form of virus replication complexes (VCRs), or in the form of viral particles (4). The capacity to reach the vascular system will allow plant viruses to spread to distal tissues of the plant. This process, known as systemic movement, involves the entry and exit of the virus from the vascular tissue. From what is known so far, most viruses use the phloem to spread systemically through the plant (5), although some cases occur via the xylem (6, 7).

Among the different mechanisms that plant viruses use for cell-to-cell movement, some of the best studied are those involving MPs of the 30K superfamily. To this MP superfamily there belong at least 18 viral genera that encode MPs related to the 30-kDa MP of tobacco mosaic virus (TMV) (8, 9). This diverse group of viral genera presents mainly two cell-to-cell movement mechanisms: (i) MPs target plasmodesmata through the cytoskeleton and probably also through the endomembrane system, modifying their size exclusion limit to allow the transport of vRNPs (4), and (ii) viral MPs form tubular structures that replace the desmotubules associated with the plasmodesmata. These tubules modify the pore size and allow the transfer of entire viral particles (4, 10). Furthermore, some members of the *Bromoviridae* family, belonging to the 30K superfamily, have both of the above characteristics: they are able to form tubules and vRNPs. Thus, some viruses, such as alfalfa mosaic virus (AMV), can move both as vRNPs and as viral particles (11–13), although when and where they use each of them are unknown.

To reach the vasculature and spread throughout the plant, viruses must spread through different cell types. In the case of viruses that move systemically through the phloem tissue, they should first colonize the mesophyll cells and then pass through the bundle sheath and vascular parenchyma, to finally reach the companion cells that connect to the phloem sieve elements (14). For the systemic movement, the entrance and exit of the vascular tissue are key and may be controlled by different molecular mechanisms that may imply viral and host proteins (15, 16).

Regarding the way in which viruses move through the phloem, it can be in the form of both virions and vRNP complexes, the latter assisted by viral proteins and/or phloem proteins (5, 17, 18). Thus, some viruses move strictly in the form of viral particles, such as some members from the *Alfamovirus*, *Closterovirus*, *Cucumovirus*, and *Potexvirus* genera, among others. Sometimes it is unknown if the movement occurs in the form of viral particles or vRNP, but the presence of the coat protein (CP) is strictly necessary (19). Although CP is associated with the formation of viral particles, in some cases it can also be part of vRNP complexes (20). Indeed, the CP could be involved in the loading and unloading of viruses in and out of the phloem (21). In addition, some viruses, such as potato mop-top virus, can move both as viral particles and as vRNP complexes (22). On the other hand, there are viruses that move in one form or another, depending on the host that they parasitize, such as tomato golden mosaic virus (23). Finally, movement can occur exclusively in the form of vRNPs. This is the case of members of the *Umbravirus* genus. The viruses that belong to this genus do not possess CP and do not form typical viral particles. These viruses move through the vasculature as RNP filament complexes (24).

Another feature to consider is in which cell type takes place the encapsidation for those viruses that move long distances in the form of viral particles and in the form of vRNPs from cell to cell. Studies with cucumber mosaic virus (CMV) have shown that the assembly occurs in the sieve elements and not in the companion cells (25).

The fact that the systemic movement of many viruses occurs in the form of vRNPs questions the role of encapsidation in long-distance movement. On the one hand, encapsidation ensures the arrival of all the genetic components at other healthy cells, where they must be expressed to ensure that the infection progresses. The encapsidation also protects the viral genome against enzymatic degradation (26, 27), although this protection could be also achieved by the vRNP formation. In addition, as far as RNA viruses are concerned, no RNase activity has been detected in the phloem (28, 29). In this context, it seems logical to speculate that encapsidation might not be necessary for the systemic movement but rather for the virus plant-to-plant transmission. According to this hypothesis, viral capsids might protect the viral genome against environmental agents during the spread of the virus to other healthy plants. This hypothesis agrees with the peculiar transmission of viruses belonging to the *Umbraviridae* family, whose long-distance transport is strictly as vRNPs, but which are spread from one plant to another encapsidated with the CP of a helper virus from the *Luteoviridae* family (24).

AMV and brome mosaic virus (BMV) are two viruses that belong to the *Alfamovirus* and *Bromovirus* genera, respectively, which are both within the family *Bromoviridae* and whose MPs belong to the 30K superfamily. Three positive-sense RNAs constitute their genome (30). RNA1 and RNA2 are monocistronic and encode subunits 1 and 2 of the viral replicase. RNA3 is dicistronic and encodes the MP and the CP. The latter is expressed through a fourth subgenomic mRNA (31, 32). Like other members of the *Bromoviridae*, AMV has a dual intercellular mechanism: it can move either as entire viral particles or as vRNPs formed by RNA, MP, and CP (33). BMV has been described as moving cell to cell as viral particles but also in the absence of the CP (12, 34, 35). Regarding systemic movement, AMV has been described as moving only as entire viral particles, while BMV can move both in the form of viral particles and in the form of vRNPs. Furthermore, the three RNAs that constitute the BMV genome may travel independently (31, 32, 36–38).

AMV MP is functionally interchangeable for short- and long-distance transport by the corresponding gene from viruses belonging to different members of the 30K superfamily. Using this system, it has been shown that MPs from viruses that move cell to cell as entire particles are also able to move as vRNPs (13, 33, 39), questioning the exact role of viral particles in virus transport.

To further identify critical aspects related to the viral systemic transport, we have designed a compensatory evolution experiment, using an AMV RNA3 derivative defective in systemic transport, to identify critical regions of the viral MP and/or CP or the viral RNA required for systemic transport. The results obtained revealed that the viral MPs have the capacity to allow long-distance movement without the requirement of virus particles.

## RESULTS

### Compensatory evolution experiment.

**FIG 1:**
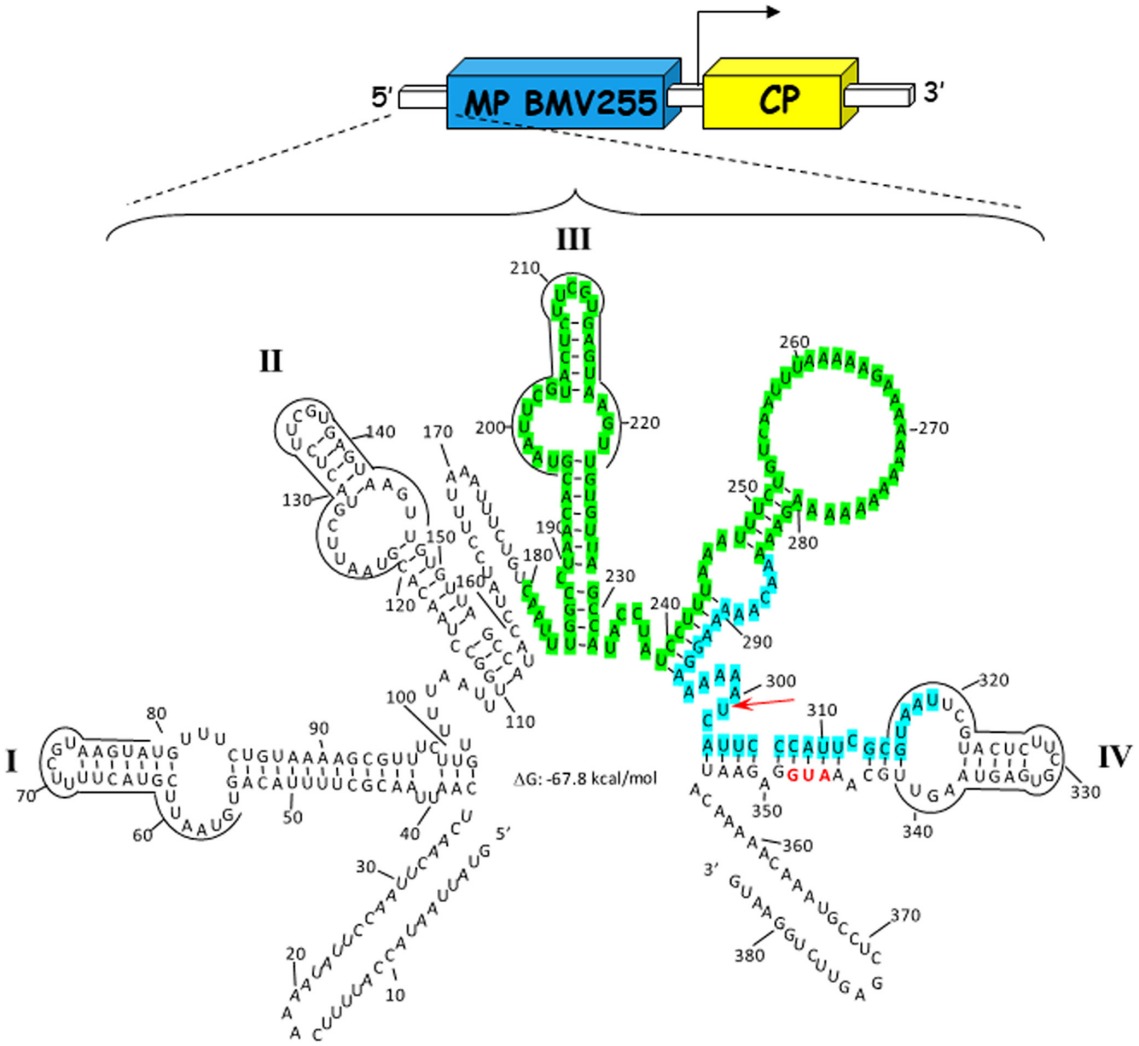
Minimal energy free structure of the 5′ UTR of AMV RNA3. The structure of the 5′ UTR was generated using mfold as implemented in the UNAFold server (40) (http://www.unafold.org/mfold/applications/rna-folding-form-v2.php) using the default settings. The minimal-energy structure contains four stem loops (I, II, III, and IV) sharing identical nucleotides (overlined) at the loop and part of the stem. An additional stem carrying a poly(A) loop was observed between stems III and IV. The scheme represents the AMV RNA3 derivative, used in the evolution experiment and unable to infect systemically the P12 plants, carrying the MP of BMV but lacking the C-terminal 48 amino acids (31). The blue and yellow big boxes represent the BMV MP and the AMV CP, respectively. The small white boxes represent the 5′ UTR and the 3′ UTR, whereas the subgenomic promoter is indicated by the arrow. In the nucleotide sequence, the BMV MP star codon is indicated in red. The three AMV constructs detected in systemic P12 leaves during the evolution experiment contained modification of the 5′ UTR consisting of a deletion of 140 nucleotides (blue and green highlighted bases) (construct 5′UTRΔ140) or a deletion of 105 nucleotides (green highlighted bases) plus the insertion of 5 (construct 5′UTRΔ105+5A) or 9 (construct 5′UTRΔ105+9A) adenines between nucleotides at positions 300 and 301 (red arrow).

Many of the different viral processes controlling systemic transport are still poorly known. In the present study, we have exploited the defective systemic transport of a chimeric AMV RNA3 construct to perform a compensatory evolution experiment designed to allow natural selection to identify critical regions of the viral RNA, MP, or CP required for proper systemic transport. In this construct, the AMV *MP* gene was exchanged with a mutated BMV *MP* gene lacking codons encoding the C-terminal 48 amino acids (31), which made it unable to interact with the AMV CP (39) (Fig. 1). Transcripts of this AMV RNA3 derivative were inoculated into five transgenic Nicotiana tabacum plants constitutively expressing the P1 and P2 subunits of the AMV RNA polymerase (P12 plants) to initiate five independent evolutionary lineages (see Fig. S1 in the supplemental material). In each passage, the systemic leaves were analyzed at 14 days postinoculation (dpi) by tissue printing the transversal section of the petiole, hybridizing the blot with a riboprobe complementary to the AMV RNA3 3′ untranslated region (3′ UTR) to look for the presence of viral progeny. At the same time, the inoculated leaves were used to inoculate the next P12 plant at 6 dpi. Between the 6th and 7th passages, we detected positive hybridization signals from the upper noninoculated leaves in all the five evolutionary lineages. Reverse transcription-PCR (RT-PCR) amplification of the complete AMV RNA3, using specific primers of total RNA extracted from the upper noninoculated leaves showing a positive hybridization signal, revealed the presence of three different AMV RNA3 variants carrying modifications only in the 5′ UTR (Fig. 1). Four of the five evolutionary lineages presented the same 5′-UTR modification consisting of a deletion of 140 nucleotides from positions 180 to 320 (construct 5′UTRΔ140). The other two AMV RNA3 variants were detected in the same evolutionary lineage and contained a deletion of 105 nucleotides from positions 180 to 285 plus the incorporation of five (5′UTRΔ105+5A) or nine (5′UTRΔ105+9A) adenines after position 300 (Fig. 1). No other modifications were observed in the AMV RNA3 hybrid, including both BMV MP and AMV CP coding sequences, indicating that mutated BMV MP is competent to transport the AMV RNA3 systemically in P12 plants without the interaction with the AMV CP. RNA folding analyses of the evolved 5′ UTR performed with mfold (40) showed the presence of the stem-loops I, II, and IV, whereas stem-loop III and the stem carrying a poly(A) loop were not present in the new 5′-UTR variants (Fig. S2). For the constructs carrying the 5′UTRΔ105+5A and 5′UTRΔ105+9A 5′ termini, no secondary structure differences were observed.

### Protoplast analysis showed that the modified 5′ UTR reduced only the accumulation of the BMV MP.

**FIG 2:**
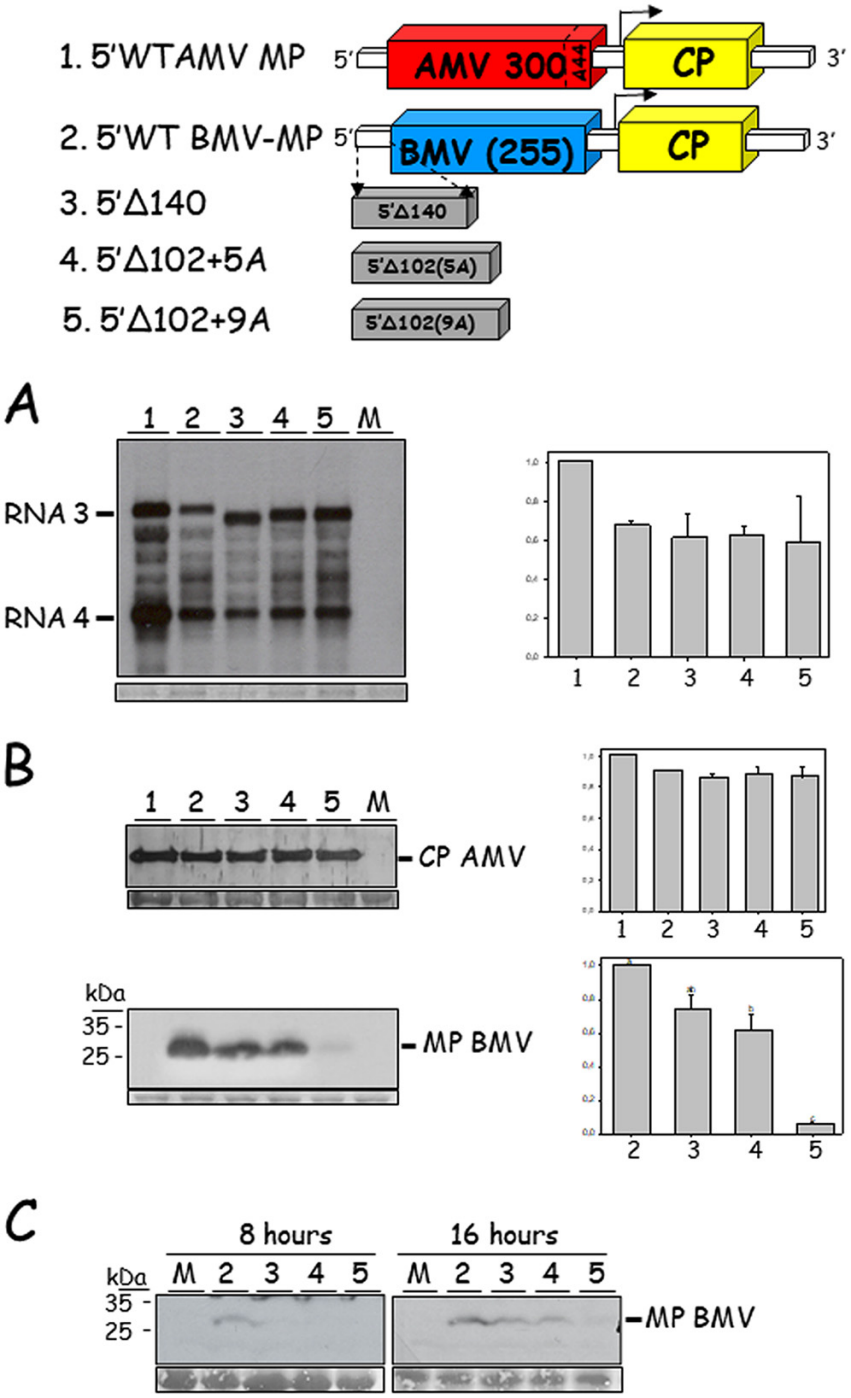
Accumulation of viral RNA, MP, and CP in P12 protoplasts transfected with the AMV RNA3 derivatives obtained in the evolution experiment. P12 protoplasts were transfected with transcripts derived from the AMV RNA3 wild type or its derivatives expressing the mutant BMV MP, lacking the C-terminal 48 amino acids, carrying the 5′UTR-WT (no. 1) construct or the 5′-UTR mutant 5′UTRΔ140 (no. 2), 5′UTRΔ105+5A (no 3), and 5′UTRΔ105+9A (no. 4) constructs obtained in the compensatory evolution experiment and described in Fig. 1. For each sample, the total amount of transfected protoplast was divided into three parts for the Northern blot analysis and the two Western blot analyses. (A) Northern blot analysis of total RNA extracted at 16 hpt using a digoxigenin-labeled RNA probe complementary to the 3′ UTR of AMV RNA3. (B) Western blot analysis of the accumulation of the AMV CP and BMV MP. The films were exposed for 30 min. (C) Time course accumulation of the BMV MP. For each sample, the protoplasts were divided into four parts corresponding to the accumulation at 2, 4, 8, and 16 hpt. No signal was detected at 2 and 4 hpt (not shown). The schemes represent the AMV RNA3 and its derivatives, where big boxes correspond to the AMV MP (red) and CP (yellow) or the BMV MP lacking the C-terminal 48 amino acids (blue). White small boxes represent the noncoding regions, and the subgenomic promoter is represented by an arrow. Numbers in the AMV and BMV MPs indicate the protein residues. The bands were quantified using the ImageJ version 2.0cr software, and the percentages refer to the 5′UTR-WT BMV-MP construct (no. 1). The results presented correspond to one of the three independent experiments. The localizations of the AMV RNA3, RNA4, AMV CP, and BMV MP are indicated in the margin. M, mock-transfected protoplasts. Graphs represent the relative accumulation of RNA3 and -4 (A) or the AMV CP and BMV MP (B) from three different experiments, in which the error bars correspond to standard deviation. Different letters above the bars indicate homogeneous groups.

To analyze the influence of the evolved 5′ UTR on the different viral infection processes, we determined the viral RNA, MP, and CP accumulation in protoplasts. Transcripts derived from the three AMV RNA derivatives obtained in the evolution experiment (5′UTRΔ140, 5′UTRΔ105+5A, and 5′UTR Δ105+9A) plus the original construct (5′UTR-WT) and the AMV RNA3 wild type (WT) were transfected to P12 protoplasts. Sixteen hours posttransfection (hpt), the transfected protoplasts were divided in three aliquots, which were used for the analysis of viral RNA progeny and MP and CP accumulation, respectively (Fig. 2). Northern blot analysis revealed that the evolved AMV constructs accumulated comparable levels of RNA3 and RNA4 compared to the original construct (5′UTR-WT BMV-MP) used to initiate the evolution experiment. The highest RNA3 and RNA4 accumulation was observed with the AMV RNA3 wild type (Fig. 2A; construct no. 1). In accordance with the observed RNA4 accumulation (responsible for the CP expression), Western blot analysis revealed no differences in the accumulation of the CP (Fig. 2B). However, the accumulation of the BMV MP (translated from the RNA3) was much lower in the evolved constructs (10 to 83%), especially in the 5′UTRΔ105+9A construct (10%) (Fig. 2B). To discard that the low BMV MP accumulation was due to a putative degradation of the protein, we performed a protoplast time course experiment, analyzing the accumulation of the BMV MP at 2, 4, 8, and 16 hpt (Fig. 2C). No detectable levels of BMV MP were observed at 2 and 4 hpt (data not shown), but at 8 and 16 hpt we observed the same MP accumulation pattern, in which the evolved AMV constructs accumulated smaller amounts of the viral MP. These results clearly indicate that the evolved 5′ UTRs negatively affected the expression of the MP.

### The evolved 5′ UTR negatively affected the encapsidation of viral RNA.

**FIG 3:**
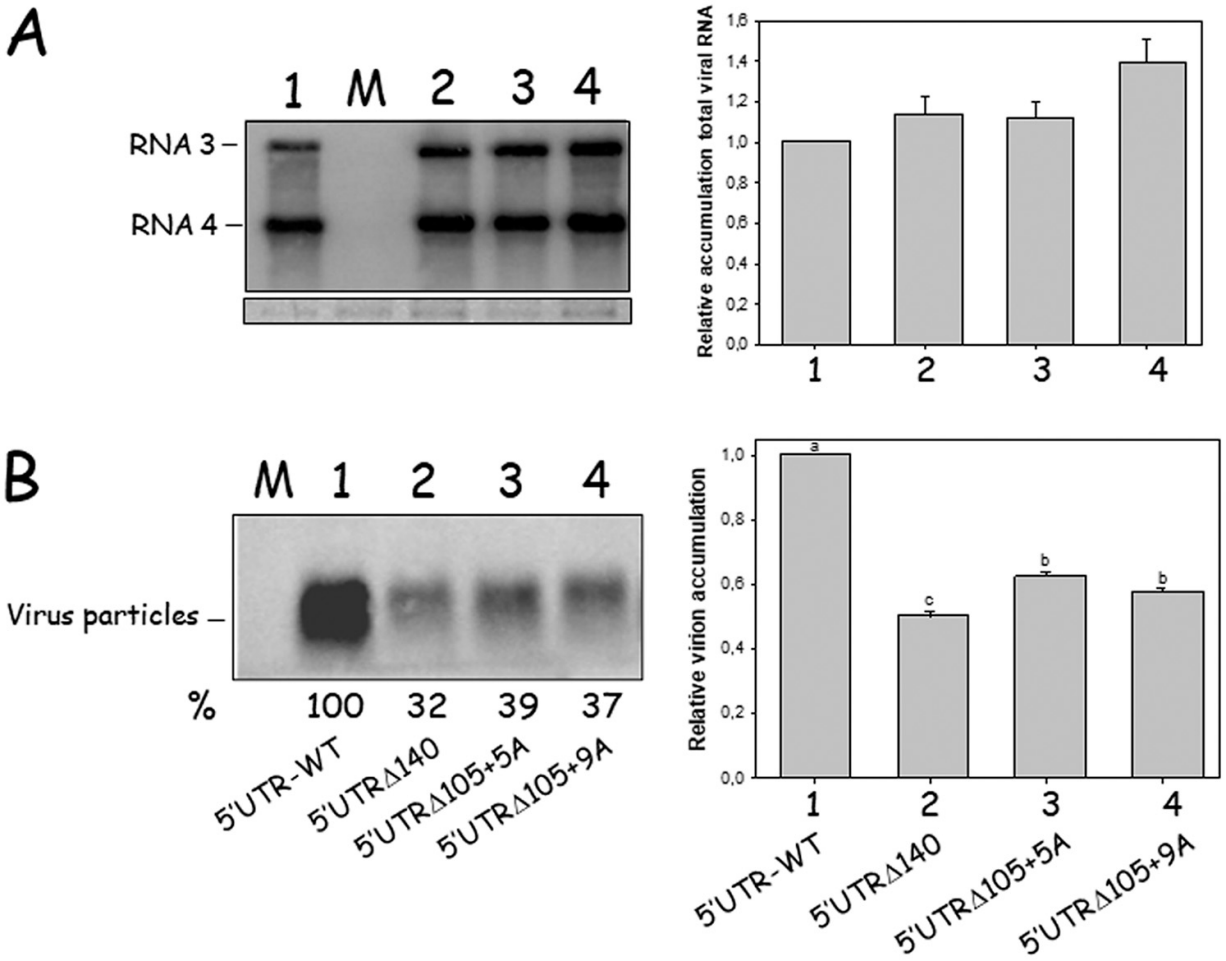
Accumulation of virus particles in P12 protoplasts. Protoplasts were transfected with transcripts derived from the AMV RNA3 derivatives indicated in Fig. 2 and carrying the BMV MP with the 5′UTR-WT (no. 1) construct or the mutant 5′UTRΔ140 (no. 2), 5′UTRΔ105+5A (no. 3), and 5′UTRΔ105+9A (no. 4) constructs obtained in the evolution experiment and described in Fig. 1. For each sample, the protoplasts were divided into two parts used for the analysis of viral progeny (A) and virus particles (B). (A) Northern blot analysis of the accumulation of viral progeny using total RNA extracted from protoplasts at 16 hpt. (B) Accumulation of encapsidated viral RNAs in protoplasts at 16 hpt. Both membranes were hybridized with a digoxigenin-labeled riboprobe complementary to the 3′ UTR of AMV RNA3. Films were exposed for 30 min. The positions of the viral RNA3, RNA4, and virus particles are indicated in the left margin. The bands were quantified using the ImageJ version 2.0cr software, and the percentages refer to the 5′UTR-WT construct (no. 1). The results presented correspond to one of the three independent experiments. Graphs represent the relative accumulation of RNA3 and RNA4 (A) or the AMV virus particles (B) from three different experiments, in which the error bars correspond to standard deviation. Different letters above the bars indicate homogeneous groups.

Virus particles carrying the viral RNA are critical for the AMV systemic transport (41). Hence, the influence of the evolved 5′ UTR on the accumulation of virus particles containing the viral RNA was analyzed in P12 protoplasts. Protoplasts were transfected with transcripts derived from the evolved AMV derivatives (5′UTRΔ140, 5′UTRΔ105+5A, and 5′UTRΔ105+9A) plus the original construct (5′UTR-WT). At 16 hpt, half of the transfected protoplasts were processed to analyze the accumulation of the viral progeny, whereas the other half were treated to analyze the accumulation of encapsidated viral RNA (Fig. 3). Northern blot analyses revealed that the accumulation of the viral progeny derived from the evolved AMV constructs (considering both RNA3 and RNA4) was comparable to that obtained from the original 5′UTR-WT construct as observed previously (Fig. 3A). However, in spite of the similar viral progeny accumulation observed for the evolved AMV constructs, the accumulation of virus particles containing the viral RNA was 32 to 39% lower, indicating that the evolved 5′ UTR negatively affects the encapsidation of viral RNAs.

### The evolved 5′ UTRs interact with the AMV CP with a reduced affinity.

**FIG 4:**
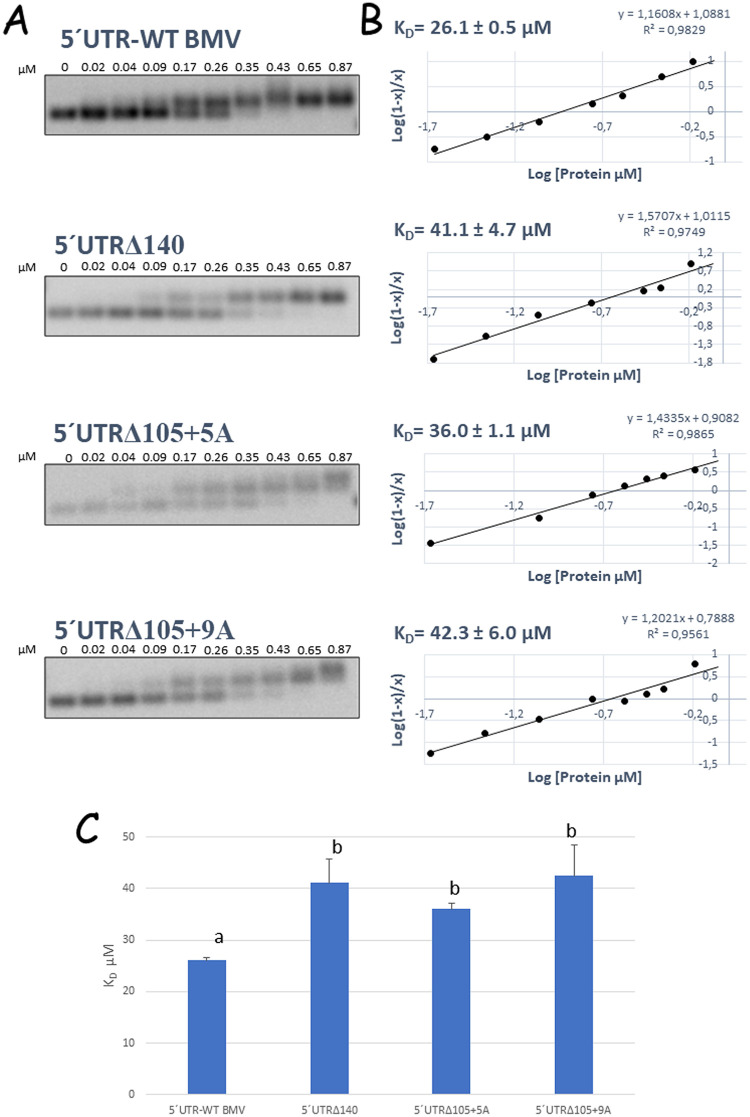
Analysis of the RNA-protein complexes formed between the AMV CP and the 5′ UTR of AMV RNA3 and its derived mutants. (A) EMSA analysis for the determination of the dissociation constant (*K_D_*) between the AMV RNA3 5′ UTR or its derived mutants and the AMV CP. Five nanograms each of the 5′ UTR of the AMV RNA3 wild-type (5′UTR-WT) or its derived 5′UTRΔ140, 5′UTRΔ105+5A, and 5′UTRΔ105+9A mutants, corresponding to RNA concentrations of 4.5 × 10^−9^ M, 7.6 × 10^−9^ M, 6.3 × 10^−9^ M, and 6.2 × 10^−9^ M, respectively, was incubated with different AMV CP concentrations (numbers on the top of the gels). The RNA-CP complexes were separated on agarose gels, transferred to nylon membranes, and hybridized with a digoxigenin-labeled riboprobe complementary to the 5′-UTR wild-type sequence. The films were exposed for 10 min. (B) Hill transformation of the RNA-protein complexes determined in panel A. The line corresponds to the best fit to a linear model, with the corresponding regression coefficient (*R*^2^) and equation given in the graph. Dissociation constants (*K_D_*) can be obtained from the linear regression equations as the intercept in the ordinate axis. (C) Graphical representation of the *K_D_* determined for the interaction of the AMV CP with the indicated 5′-UTR AMV RNA3. The error bars correspond to standard deviations from three different EMSAs. Different letters above the bars indicate homogeneous groups.

The reduced encapsidation observed with the AMV RNA variants carrying the evolved 5′ UTRs points to the idea that this region could be affecting the interaction of the viral RNA with the AMV CP during the encapsidation process. In the next step, the capacity of the 5′ UTR to interact with the AMV CP was analyzed by an electrophoretic mobility shift assay (EMSA). To do this, 5 ng of transcripts corresponding to the 5′ UTR of the AMV RNA3 wild type or the three evolved 5′ UTRs was incubated with different concentrations of purified AMV CP carrying a N-terminal histidine epitope (37). The estimation of the *K_D_* (dissociation constant) values from three different experiments revealed a value of 26.1 ± 0.5 μM for the 5′ UTR wild type, which was increased by 57.4% (41.1 ± 4.7 μM), 37.9% (36.0 ± 1.1 μM), and 62.0% (42.3 ± 6.0 μM) for the 5′UTRΔ140, 5′UTRΔ105+5A, and 5′UTRΔ105+9A mutants, respectively (Fig. 4). The mutated 5′ UTRs clearly reduced the affinity of the AMV CP, which correlated with the reduced encapsidation process observed in the protoplast experiments.

### The evolved 5′ UTR increased cell-to-cell transport.

**FIG 5:**
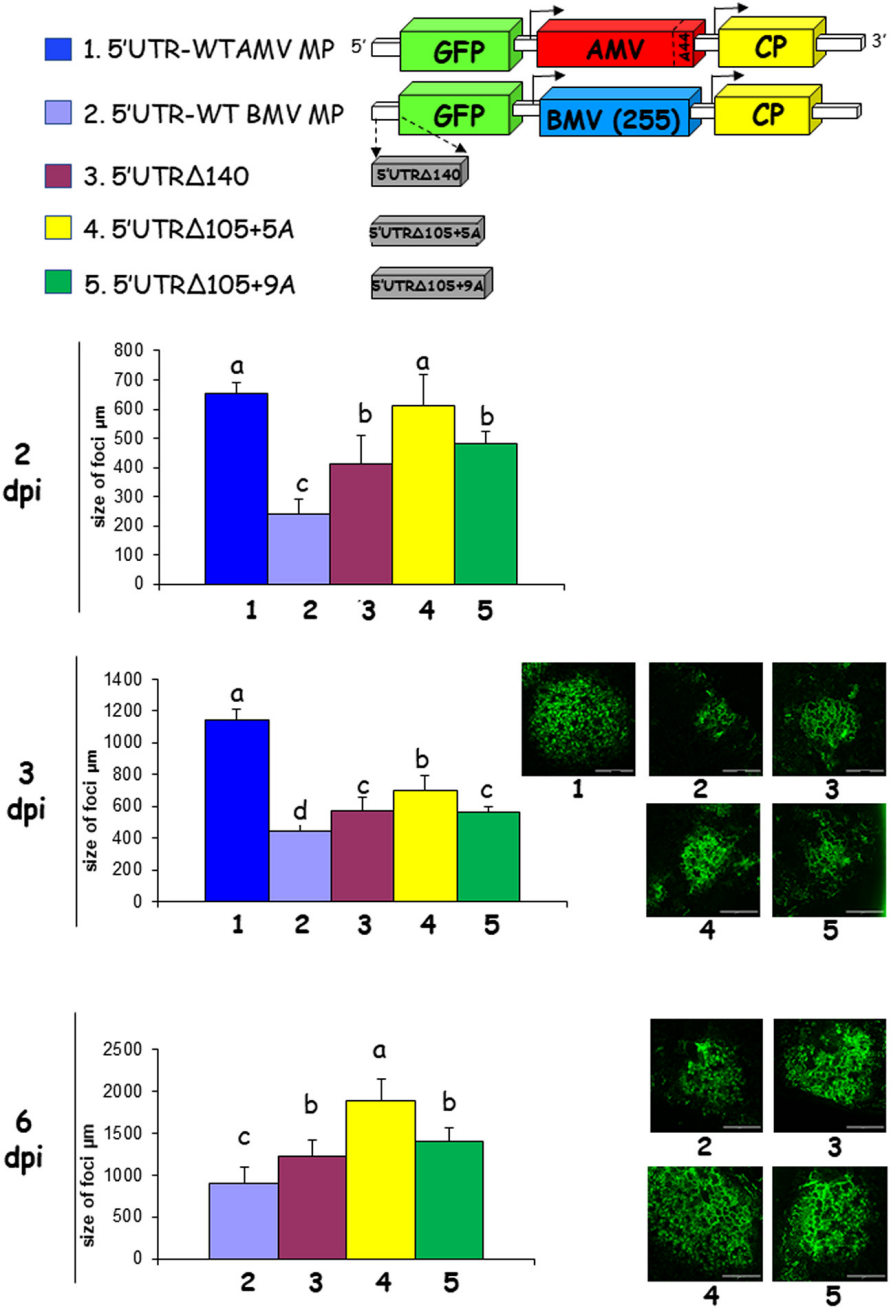
Effect of the 5′ UTR in the cell-to-cell movement of the AMV RNA3 derivatives. P12 plants were inoculated with transcript derived from the wild-type AMV RNA3 construct expressing GFP (61) and its derivatives carrying the BMV MP with the 5′UTR-WT (no. 1) construct or the mutant 5′UTRΔ140 (no. 2), 5′UTRΔ105+5A (no. 3), and 5′UTRΔ105+9A (no. 4) constructs obtained in the compensatory evolution experiment and described in Fig. 1. Graphs represent the perimeter of 20 infection foci at 2, 3, and 6 dpi. Error bars correspond to standard deviation. Different letters above the bars indicate homogeneous groups. Different numbers of asterisks indicate significant cell-to-cell differences, while similar numbers of black asterisks indicate no significant cell-to-cell differences. Schemes represent the AMV RNA3 construct expressing GFP and its derivatives, where big boxes indicate the open reading frames of GFP (green), AMV MP (red), AMV CP (yellow), and BMV MP (blue). Images correspond to infection foci representative of each indicated AMV construct. Bars correspond to 200 μm.

**FIG 6:**
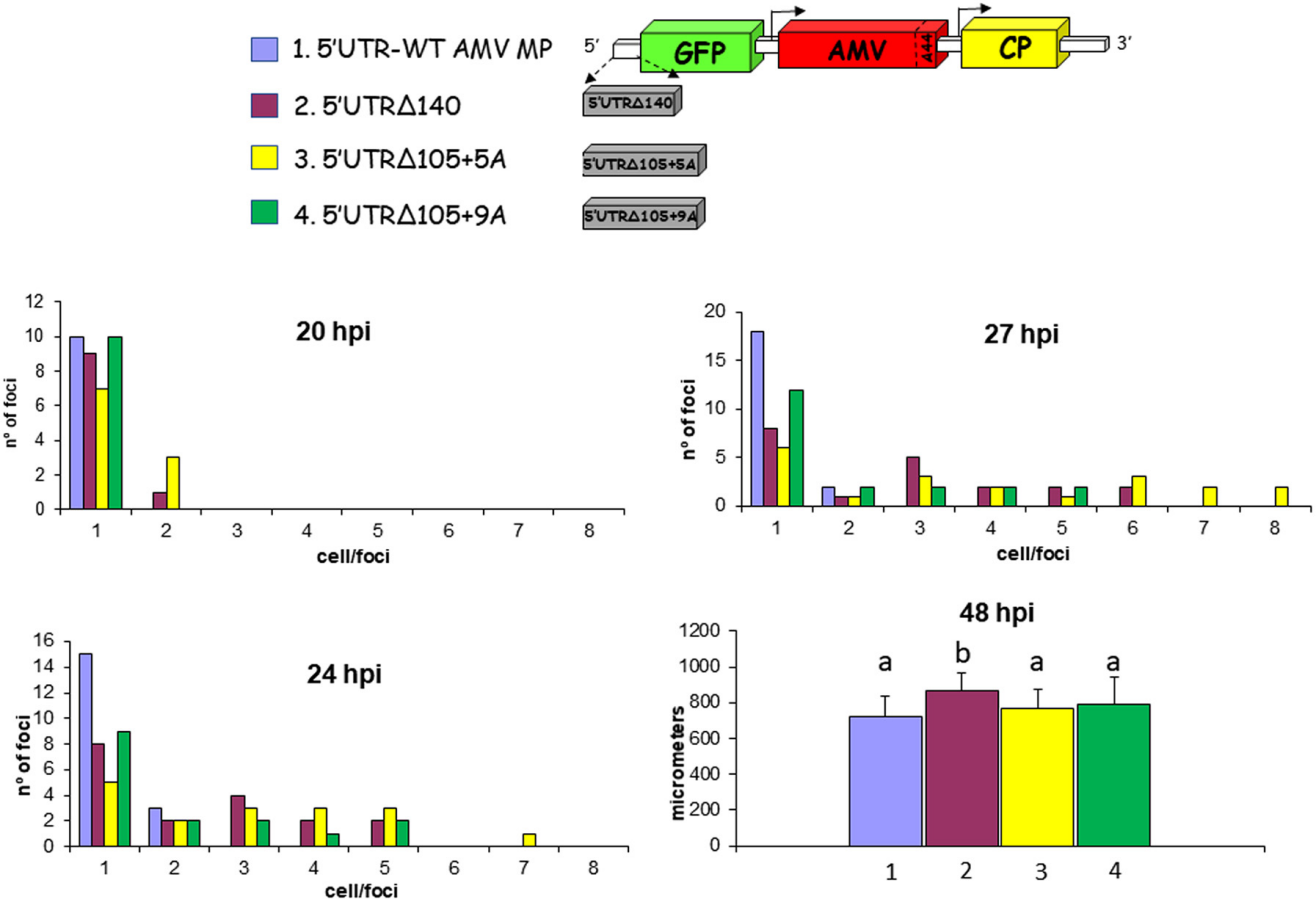
Influence of the 5′ UTR on the cell-to-cell transport of AMV RNA3 wild type. P12 plants were inoculated with transcripts derived from the AMV RNA3 construct expressing GFP and carrying the 5′UTR-WT (no. 1) construct (61) or the mutant 5′UTRΔ140 (no. 2), 5′UTRΔ105+5A (no. 3), and 5′UTRΔ105+9A (no. 4) constructs obtained in the compensatory evolution experiment described in Fig. 1. Graphs represent the number of fluorescent cells per infection focus at 20, 24, and 27 hpi or the perimeter of 20 infection foci at 48 hpi. Different letters above the bars indicate statistically homogeneous groups. The scheme represents the AMV RNA3 construct expressing GFP as indicated in Fig. 4.

Previous analysis performed with the AMV RNA3 carrying different MPs of the 30K superfamily, suggested the requirement of a minimal cell-to-cell invasion capacity to reach the vascular tissue and, consequently, invade the upper noninoculated tissue (33). Next, we evaluated the influence of the evolved 5′ UTR on cell-to-cell transport. To do that, we used an AMV RNA3 derivative carrying the green fluorescent protein (GFP) plus the mutated BMV MP (31) and the different 5′ UTRs. Transcripts derived from the different AMV constructs plus the AMV wild type (containing the AMV MP) were inoculated into P12 plants, and the perimeter of 20 infection foci was measured at 2, 3, and 6 dpi (Fig. 5). The results obtained revealed that the infection foci derived from the constructs carrying the evolved 5′ UTR (5′UTRΔ140, 5′UTRΔ105+5A, and 5′UTRΔ105+9A) were significantly larger than the original construct carrying the 5′UTR-WT BMV MP on all analyzed days postinfection (Fig. 5). No significant differences were observed between the 5′UTRΔ140 and 5′UTRΔ105+9A constructs, showing similar infection foci perimeter on all days. The bigger infection foci were observed with the 5′UTRΔ105+5A construct, showing a similar size of infection foci to that observed from the AMV wild type (5′UTR-WT AMV MP) at 2 dpi, but significantly lower at later dpi. Altogether, these results indicate that the evolved 5′ UTRs increased the cell-to-cell transport of the viral RNA and also that the number of inserted adenines is critical for this process, with a significant difference observed in the infection focus perimeters at 2 dpi between the 5′UTRΔ105+9A and 5′UTRΔ105+5A constructs, which differed only in the presence of four adenines. To see if the evolved 5′ UTR also incremented the cell-to-cell transport of other AMV derivatives, we analyzed its influence on the AMV RNA3 wild type (Fig. 5). Transcripts derived from the AMV construct carrying the AMV MP, GFP, and the 5′-UTR wild type (5′UTR-WT AMV MP) or the different evolved 5′ UTRs, were inoculated into P12 plants. The analysis of the infection focus perimeter observed after 2 dpi revealed no significant differences between all assayed constructs (data not shown). Accordingly, we decided to analyze the cell-to-cell transport at shorter incubation times by counting the numbers of infected cells per focus at 20, 24, or 27 h postinoculation (hpi) or by determining the infection focus perimeter at 48 hpi. At 20 hpi, we observed only single infected cells for all constructs, except the AMV derivatives carrying the 5′UTRΔ140 and 5′UTRΔ105+5A constructs, which presented a reduced number of foci with two fluorescent cells. When the same inoculated plants were analyzed at 24 or 27 hpi, we observed that all AMV derivatives carrying the evolved 5′ UTRs presented infection foci containing up to five or six infected cells (seven or eight cells in the case of the 5′UTRΔ105+5A construct). In contrast, the AMV construct carrying the 5′UTR-WT construct presented mainly single infected cells or a reduced number of foci with two cells (Fig. 6; 24 and 27 hpi). In spite of the observed differences at short incubation times, the analysis of the infection focus perimeter at 48 hpi revealed no significant differences between all analyzed constructs, except for the 5′UTRΔ140 derivative (Fig. 6; 48 hpi). Further increase of the infection time (3 dpi) rendered similar infection foci for all constructs (data not shown). Apparently, the positive cell-to-cell increase derived from the evolved 5′ UTR is also present in the AMV RNA3 wild type, but only at early infection times, indicating that other factors (e.g., the AMV MP or its capacity to interact with the CP) could compensate for such an effect.

### The evolved 5′ UTR permits systemic transport of AMV in the absence of virus particles.

**FIG 7:**
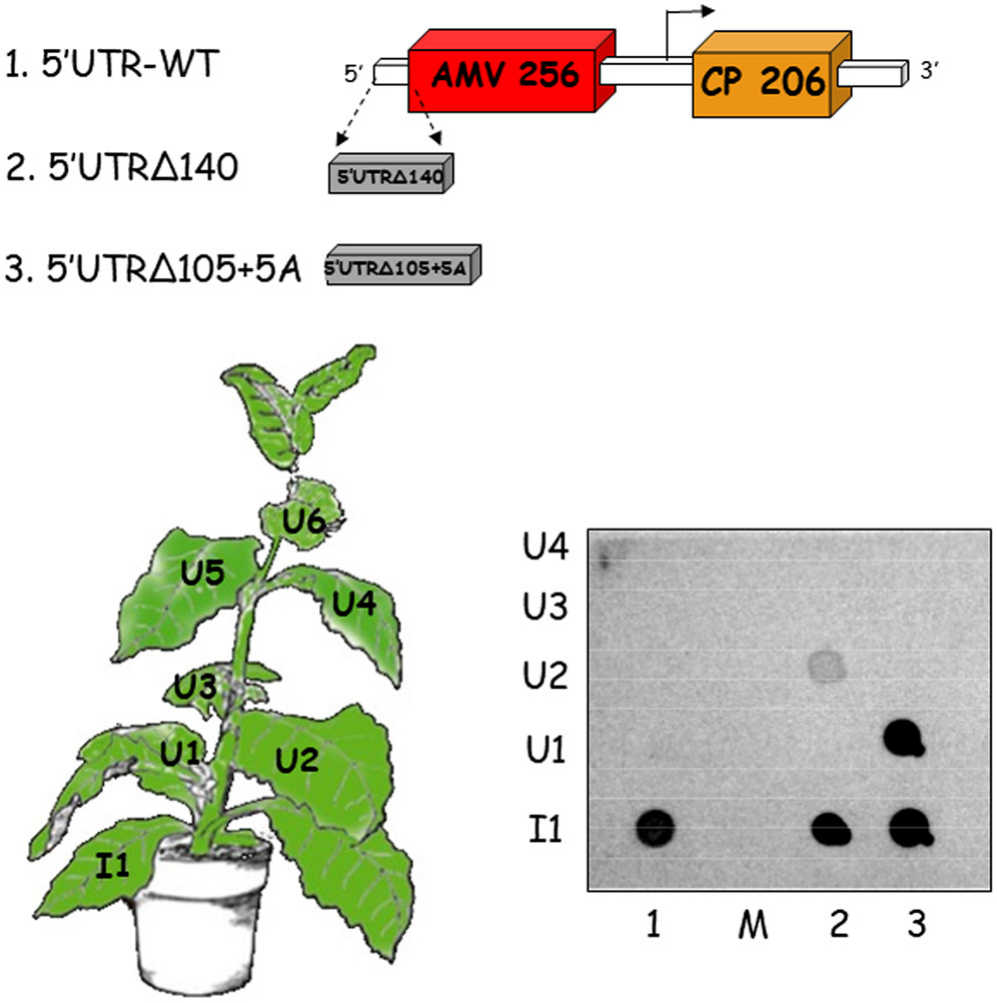
Influence of the 5′ UTR on the systemic transport of a virion-defective AMV RNA3 mutant. P12 plants were inoculated with transcripts derived from an AMV RNA3 mutant expressing a CP lacking the C-terminal 14 residues (CP 206) and unable to generate virus particles and an MP lacking the C-terminal 44 residues (MP 256), required for the specific interaction with the cognate CP. The analysis was performed with three variants of the same construct carrying the 5′UTR-WT (no. 1) construct or mutant 5′UTRΔ140 (no. 2) and 5′UTRΔ105+5A (no. 3) constructs obtained in the compensatory evolution experiment and described in Fig. 1. At 12 dpi, the inoculated plants were analyzed by printing the transversal section of the petiole of all inoculated (I) and upper (U) noninoculated leaves. The membranes were hybridized with a digoxigenin-labeled riboprobe complementary to the 3′ UTR of the AMV RNA3. The scheme corresponds to the AMV RNA3 as indicated in Fig. 2. Numbers in the MP and CP genes indicate the amino acids of the expressed proteins. The precise localization of the analyzed leaves is indicated in the plant scheme. M, mock-inoculated plant.

Previous results showed that an interaction between the C terminus of AMV MP and CP could be required for the virus transport (31). The results reported above in which the truncated BMV MP, deficient in the interaction with the AMV CP, is able to support the systemic transport of AMV RNA3 when the 5′ UTR evolved to shortened versions argue against this requirement and, on the other hand, strongly suggest that virus particles may not be required for the AMV long-distance transport. To confirm this observation, we analyzed the effect of the evolved 5′ UTR in an AMV RNA3 derivative carrying a mutated CP defective in virus particle formation (CP206) (41) and also a mutated MP lacking the C-terminal 44 amino acids (AMV 256) involved in the specific interaction with CP (39) (Fig. 7). Transcripts derived from the AMV RNA3 construct carrying the 5′-UTR wild type or the evolved 5′UTRΔ140 and 5′UTRΔ105+5A 5′ termini, were inoculated into P12 plants. The presence of the AMV progeny in the upper noninoculated leaves was analyzed at 14 dpi. Dot blot analyses revealed positive hybridization signals in all inoculated leaves; however, positive hybridization signals in the upper noninoculated leaves were detected only in the plants inoculated with the constructs carrying the two evolved 5′ UTRs (Fig. 7). Altogether, these results indicate that neither the virus particles nor the AMV MP-CP interaction is required for the AMV systemic transport but also that the 5′ UTR plays a critical role in the AMV movement.

### The evolved 5′ UTR increased the systemic transport of AMV derivatives carrying different MPs of the 30K superfamily.

**FIG 8:**
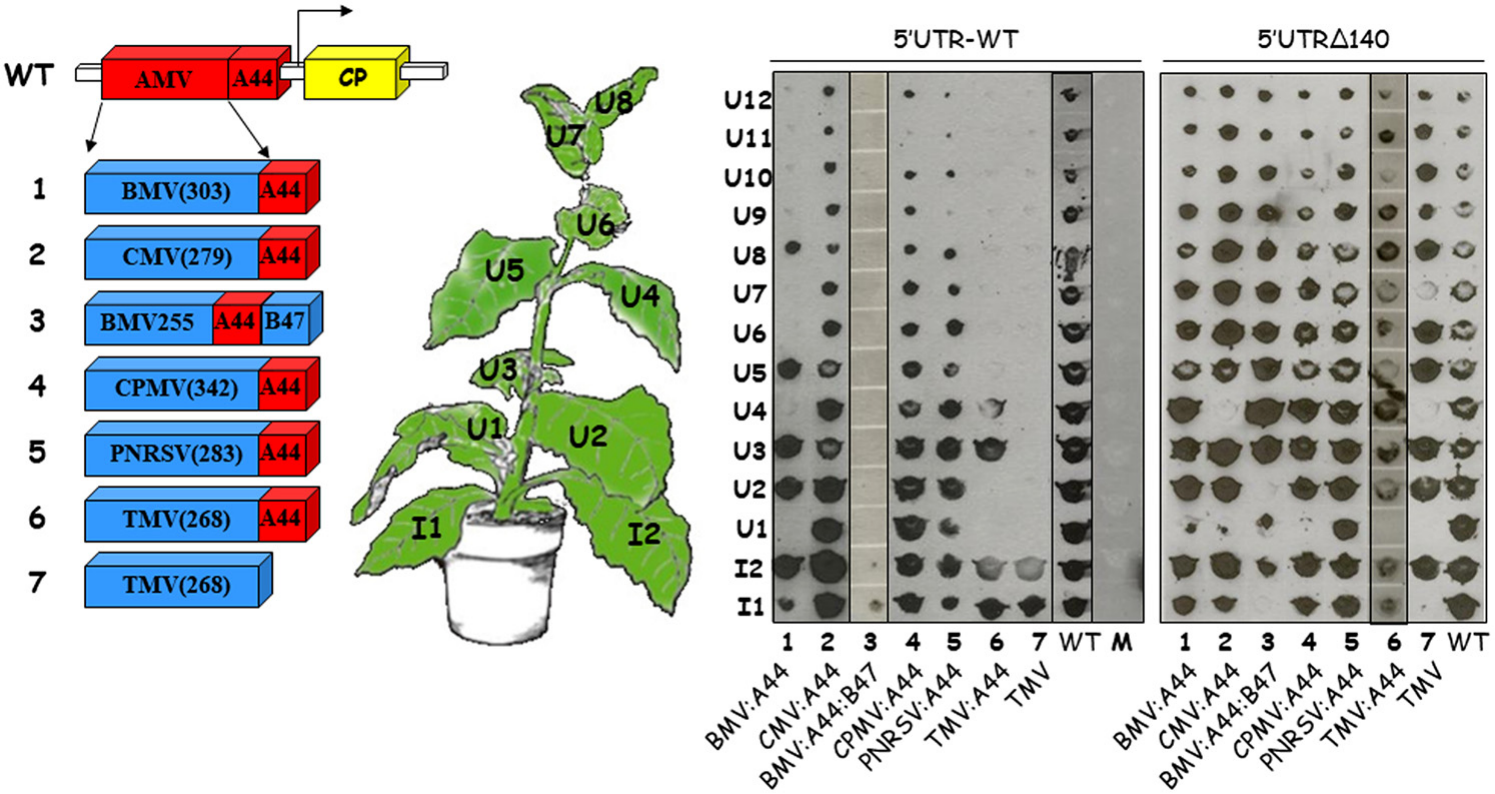
Influence of the 5′ UTR on the systemic transport of AMV RNA3 derivatives carrying different MPs of the 30K superfamily. The scheme represents the AMV RNA3 wild type and its derivatives in which the AMV MP was exchanged with the MP of BMV (no. 1 and 3), CMV (no. 2), CPMV (no. 4), PNRSV (no. 5), and TMV (no. 6 and 7) (33). The C-terminal 44- and 48-amino-acid fragments of the AMV and BMV MP are indicated as A44 and B48, respectively. The P12 plants were inoculated with transcripts derived from the AMV hybrids carrying the 5′UTR-WT or 5′UTRΔ140 mutant construct as indicated in Fig. 1. The inoculated plants were analyzed at 14 dpi by tissue printing of the inoculated leaves (I), and noninoculated upper leaves (U). The localization of the different leaves is indicated in the plant scheme. The membranes were hybridized with a DIG-labeled probe complementary to the AMV RNA3 3′ UTR. The films were exposed for 30 min. The blot corresponding to the AMV constructs carrying the 5′UTR-WT construct is reproduced from reference 33 with permission of the Society for General Microbiology. The black lines in the blot indicate different blots joined together.

Previous analysis performed with the AMV RNA3 showed the functional exchangeability of different MPs of the 30K superfamily for the local (39) and systemic (33) AMV transport. In these previous studies, the MPs of BMV, cowpea mosaic virus (CPMV), CMV, prunus necrotic ringspot virus (PNRSV), and TMV were included. In all five cases, the presence of the C-terminal 44 residues of the AMV MP (A44) conditioned the capacity of the heterologous MP to support either the local or the systemic transport. In all cases, except for the TMV MP, this A44 region was required for the cell-to-cell transport, but all analyzed MPs required its presence at the C terminus for the systemic transport. To further characterize the role of the evolved 5′ UTR in AMV systemic transport, transcripts derived from the AMV derivatives carrying different 30K MPs and containing the mutated 5′UTRΔ140 termini were inoculated into P12 plants and the presence of the viral progeny in all inoculated (I) and upper noninoculated (U) leaves was analyzed at 14 dpi by tissue printing (Fig. 8; 5′UTRΔ140). All constructs were detected in the majority of the upper noninoculated leaves, as observed for the AMV RNA3 wild type. Especially relevant were the results obtained with the AMV constructs carrying the TMV MP without the A44 region or the BMV MP with A44 preceding its C-terminal 47 amino acids (Fig. 8; constructs 3 and 7). Both constructs, carrying the 5′-UTR wild type, were detected only in the petiole of the inoculated leaves (Fig. 8; 5′UTR-WT blot). However, the presence of the 5′UTRΔ140 construct allowed the invasion of the majority of the upper noninoculated leaves (Fig. 8; 5′UTRΔ140 blot). A similar result was observed for the AMV derivatives carrying the BMV or TMV MPs with the A44 region fused at its C terminus, where the presence of the evolved 5′UTRΔ140 allowed the invasion of all noninoculated leaves, whereas the 5′UTR-WT only allowed a local spread. Altogether, these results indicate that the 5′ UTR plays a critical role in systemic AMV transport.

## DISCUSSION

In spite of the substantial information related to the cell-to-cell transport of plant viruses, little is known about the requirements for systemic infection. CP could be crucial in the infection of the specific cells located between the mesophyll cells and the sieve elements, and vice versa, during the virus entry into and exit from the vascular tissue (15). However, many other factors could be critical during the invasion of the vascular cells, including the presence of silencing suppressor, the movement proteins, and also the presence of viral proteins in the nucleus/nucleolus (37, 42–44). Regardless of the critical participation of CP in viral systemic transport, the observation of plant viruses that move systemically without the requirement of the CP (e.g., *Umbravirus*, *Pomovirus*, etc.) clearly questioned the exact role of this protein in virus transport. On the opposite side, the CP is critical for the systemic transport of viruses such as AMV. The question that arises is what precise role does the AMV CP play in viral systemic transport? In the present work, we have addressed this question by taking an experimental compensatory evolution approach, using the AMV/P12 system and a chimeric AMV RNA3 defective in systemic movement (31), to look for critical sequences of the viral RNA or regions of the expressed C-terminal BMV MP mutant and/or AMV CP required for recovery of systemic transport. The incapacity of the truncated BMV MP to interact with the AMV CP indicates that the viral RNA (vRNA) transported through the infected cells should be a complex containing the vRNA and the truncated MP, although the presence of other viral (e.g., CP) and/or host proteins could not be ruled out.

The analysis of the evolved viral progeny revealed that only modifications at the 5′-UTR sequence were sufficient to recover the systemic transport of the chimeric AMV RNA3, with no other modifications in both the BMV MP and AMV CP. Interestingly, the BMV MP was sufficient to support both the short- and the long-distance transport of viral AMV RNA3 without the interaction with an encapsidation-competent CP, a prerequisite described for this MP in the BMV context (45), indicating that BMV MP possess all required functions to support the systemic transport. However, we cannot rule out the possibility that the C-terminally truncated BMV MP had increased its RNA affinity, allowing the transport of vRNP in the absence of CP as described for the MP of CMV (11, 46). The analysis of the evolved AMV RNA3 progeny revealed comparable levels of RNA progeny and MP (at least for constructs carrying the evolved 5′UTRΔ140 and 5′UTRΔ105+5A 5′ termini) and CP expression but significantly reduced accumulation of encapsidated RNA and increased cell-to-cell transport compared to the initial AMV construct. EMSA analysis revealed that the evolved 5′ UTRs have less affinity with the AMV CP, increasing the *K_D_* between 37% and 62%, which correlated with their low accumulation of encapsidated RNAs. In this sense, the accumulation of the encapsidated AMV RNAs 3 and 4 correlated with the presence of a 5′-leader sequence of different AMV strains (47). The *K_D_* estimated for the 5′-UTR wild type was 52-fold higher than that determined for the 3′ UTR (26.1 μM versus 0.5 μM) (48). The AMV CP–3′-UTR interaction is required for the replication, translation, and transport viral processes, but not for the encapsidation (49). In this sense, the lower CP affinity observed for the 5′ UTR could represent a way to prioritize different viral processes—in this case, the encapsidation versus replication and/or translation—although we cannot discard the possibility that other parts of the viral genome are required for virus particle formation. Although the sequence required to initiate virion assembly is unknown in AMV, sequences in the 5′ UTR of other RNA viruses have been implicated in the formation of virions (50).

The analysis of the accumulation of the BMV MP revealed that the modification of the 5′ UTR also affected the translation of the viral protein, which was especially appreciable comparing the constructs carrying the evolved 5′UTRΔ105+5A and 5′UTRΔ105+9A, in which the presence or absence of four adenines is critical for protein accumulation (10% versus 83%). The observation that both constructs were present in the same infected plant permits the hypothesis that not only is this AMV derivative still evolving, probably by increasing the MP accumulation, but also reduced MP accumulation is still compatible with an efficient virus transport (Fig. 5 and 6), opening up questions about the minimal MP accumulation required for virus transport. Further analysis will be addressed to analyze the influence of the MP accumulation on short- and/or long-distance transport.

Considering the incapacity of the BMV MP to interact with the AMV CP, and as a consequence with the virus particles, we can expect that all encapsidated AMV RNAs will not be accessible to the BMV MP. In this sense, the reduced accumulation of encapsidated RNAs in the evolved chimeric AMV constructs will permit increase in the number of BMV MP-RNA complexes, allowing for more efficient virus transport. Accordingly, all evolved chimeric AMV constructs showed increased cell-to-cell transport, which is critical for the systemic transport (33), probably by avoiding the plant defense mechanisms (21, 51–54). Interestingly, a BMV MP mutant lacking the C-terminal 42 amino acids was competent to mediate cell-to-cell transport of BMV in the absence of CP, but did not support systemic transport, even in the presence of the CP (36), probably due to its incapacity to interact with the virus particles and/or the encapsidated viral RNAs, as observed herein. Deletion of the C-terminal 33 amino acids of the CMV MP also resulted in CP-independent cell-to-cell movement, but not long-distance movement (55). However, the lack of the CP in the CMV infection indicated that other factors, different from the inaccessibility of the mutated CMV MP to the encapsidated RNAs, could be critical for the systemic transport in the CMV infection. All of these results point to the idea that the system has evolved to reduce the accumulation of encapsidated viral RNA to increase the BMV MP-RNA complexes, but we cannot discard that the modifications observed at the 5′ UTR are also a consequence of the adaptation of the AMV system to the foreign BMV MP.

The evolved 5′ UTR of AMV RNA3 also increased the systemic transport of other chimeric AMV RNA3 carrying different viral MPs assigned to the 30K superfamily, including the TMV MP lacking the C-terminal 44 amino acids of the AMV MP, which is responsible to the compatible interaction with the AMV CP. In the case of the AMV MP, the presence of the evolved 5′ UTR allowed the systemic transport of viral progeny with a mutated AMV MP lacking the CP-interacting region (A44) and in the absence of encapsidated viral RNAs, indicating that virus particles and/or the MP-CP complex, reported to be critical for the systemic transport (31, 37, 41), is not needed for the virus transport. Recently, it has been observed that the MP of citrus leprosis virus C2 was also able to support AMV systemic transport in the absence of the A44 residues (44), indicating both that this MP transports infective viral complexes regardless of the interaction with the CP and also that virus particles are not required for the AMV systemic transport. Altogether, these observations permit us to hypothesize that viral MPs are competent to allow the local and systemic transport without the CP, as reported for the viruses lacking a CP (e.g., *Umbravirus*), but viruses that express a CP have probably evolved to ensure the presence of virus particles, critical for vector virus transmission, by strong CP *cis*/*trans*-elements and/or their multifunctional properties (26, 56, 57). In this sense, the AMV CP is required not only for virus transport but also for protein expression, virus infectivity, and inhibition of host defense mechanisms (58, 59). The more extreme CP dependence could be represented by the DNA viruses (e.g., *Caulimovirus*) in which the transported complex, for either local or systemic transport, is encapsidated DNA (13, 60).

In summary, we have shown that virus particles are not required for the AMV systemic transport but also that the 5′ UTR plays a critical role in virus encapsidation and, consequently, in the viral transport. Regarding the functional exchangeability observed between the MPs assigned to the 30K superfamily, it is tempting to speculate that members of this family are competent to allow the cell-to-cell and long-distance transport without the requirement of the virus particles, at least for the RNA viruses. Further analysis will be addressed to confirm this idea.

## MATERIALS AND METHODS

### Construction of infectious clones.

Constructs corresponding to the evolved AMV RNA3 derivatives carrying BMV MP, lacking the C-terminal 48 residues (31), were amplified by reverse transcription-PCR (RT-PCR) (Invitrogen SuperScript III one-step RT-PCR system; Thermo Fisher Scientific, Inc.) using total RNA extracted from the different P12 plant lines and using the sense primer VP668, corresponding to the 5′-terminal 17 nucleotides of the AMV RNA3 preceded by the T7 promoter sequence and a HindIII restriction site (5′-ACGTTAAGCTTAATACGACTCACTATAGTATTAATACCATTTTC-3′), and the antisense primer VP570, complementary to the 3′ termini of AMV RNA3 plus a PstI restriction site (5′-CACCTGCAGCATCCCTTAGGGGCATTC-3′). The resulting PCR fragments were digested with HindIII-PstI enzymes and cloned in the pUC18 plasmid, previously digested with the same restriction enzymes. The resulting constructs contain a HindIII site preceding the T7 promoter sequence and an NcoI site at the start codon of the BMV MP. Both restriction sites were used to exchange the 5′ UTR between the evolved constructs and the AMV RNA3 wild type (plasmid pAL3NcoP3 in reference 47), an AMV RNA3 wild type modified to express the GFP (61), and several AMV RNA3 chimeras carrying the previously described heterologous MP (33).

The determination of the nucleotide sequence of the different constructs generated in this project was done by the Sequencing Service of the IBMCP (CSIC-UPV) using the corresponding primers and the Sanger method.

### Protoplast preparation and inoculation.

Protoplasts were obtained from young plants (no more than 4 leaves) of N. tabacum that constitutively express the P1 and P2 subunits of AMV replicase (P12 plants). The epidermis of the selected leaves was removed by spreading carborundum on the underside of the leaf with a cotton ball. The leaves were then placed on an enzyme solution (0.4 M mannitol, 0.44% MS medium, 0.1% pectolyase, 1% cellulose [pH 5.5]) for 2 to 4 h at room temperature. After this time, the solution was shaken in order to separate the cells and filtered with Miracloth cloth in test tubes. At the bottom of the tubes, 1 mL of 20% sucrose was deposited in order to form two phases. The tubes were then centrifuged at 800 rpm for 10 min at 4°C to separate the entire protoplasts from the damaged ones. The entire protoplasts, accumulated at the interface, were placed in a new tube, which was filled with a wash solution (0.4 M mannitol, 0.44% MS medium [pH 5.6]). The sucrose gradient separation process described above was repeated, and the protoplasts were placed in ready-to-inoculate tubes with ca. 200,000 protoplasts per sample.

For protoplast inoculation, the supernatant was removed and 15 μL of the transcription reaction mixture (corresponding to 5 to 7 μg of RNA) generated with the different AMV RNA3 derivatives was mixed with 15 μL of the 2× inoculation buffer (0.8 M mannitol, 0.88% MS medium, 12 mM CaCl_2_ [pH 5.6]) and added directly to the protoplasts. The mixture was gently stirred for about 10 s to resuspend the protoplast pellet, and then 100 μL of the transfection solution (40% polyethylene glycol [PEG], 6 mM CaCl_2_) was added. Then, 4 mL of washing solution was added. After allowing the protoplasts to stand for 10 min at 4°C, they were collected by centrifugation for 3 min at 800 rpm, the supernatant was removed, and 1 mL of incubation solution was added (wash solution containing 2% sucrose). The protoplasts were kept with this solution at 25°C and under constant light for at least 16 h.

### Inoculation of P12 plants.

The transcripts corresponding to the different AMV RNA3 derivatives were obtained by transcription reactions using the T7 RNA polymerase (TaKaRa Bio, Inc.). The reactions were performed in a final volume of 20 μL containing: 2 μL 10× transcription buffer (Roche Diagnostics GmbH, Mannheim, Germany), 2 μL 10 mM nucleoside triphosphates (NTPs), 0.3 μL RNase inhibitor (40 U/μL) (Ribolock small interfering RNA [siRNA]; Thermo Fisher Scientific), 0.4 μL T7 RNA polymerase (50 U/μL) (TaKaRa Bio, Inc.), and 200 ng of the PCR-amplified DNA template. The PCR products used in the transcription reaction were generated using the sense primer 668, corresponding to the 5′-terminal 17 nucleotides of the AMV RNA3 preceded by the T7 promoter sequence (5′-ACGTTAAGCTTAATACGACTCACTATAGTATTAATACCATTTTC-3′), and the antisense 1602 primer, complementary to the 3′ terminal of AMV RNA3 (5′-GCATCCCTTAGGGGCATTCATG-3′). The inoculation of transcripts was performed using carborundum and 10 μL of transcription reaction mixture per leaf.

### Visualization and quantification of infection foci in inoculated leaves.

The use of the AMV RNA3 variant that expresses the green fluorescence protein (GFP) allowed visualization of the infection foci in infected tissue. GFP was detected at 1 (single infected cells) or 3 (infection foci) dpi using a Leica MZ 16F loupe (excitation of 488 nm and fluorescence emission in the 510- to 560-nm spectrum). The diameter of the infection foci was determined using the ImageJ program (http://rsbweb.nih.gov/ij) and 20 foci per construct.

### Analysis of inoculated P12 protoplast and plants.

Total RNA was extracted from protoplast at 16 hpi or from P12 leaves using TRIzol reagent (Thermo Fisher Scientific). The RNA was denatured by formaldehyde treatment and analyzed by Northern blotting hybridization as described previously (62) using a digoxigenin (DIG)-riboprobe (Roche Diagnostics GmbH, Mannheim, Germany) complementary to the 3′ UTR 138 of the AMV RNA3. The membranes were exposed to Kodak X-Omat AR film for ~15 min. Alternatively, the chemiluminescent signal was detected using the LAS 3000 Imaging System from Fuji.

Encapsidation of AMV RNAs into virus particles was analyzed by sedimenting 7 × 10^4^ protoplasts by centrifugation. The protoplasts were resuspended in 100 mL of PE buffer (0.01 M NaH_2_PO_4_, 1 mM EDTA [pH 7.0]), homogenized, and incubated for 30 min at room temperature to degrade nonencapsidated RNAs. RNA was extracted from the homogenate and analyzed as described above.

Tissue printing analyses were performed by transversal sections of the corresponding petiole from inoculated (I) and upper (U) P12 leaves at 14 dpi, as previously described (13). All results shown from tissue printing are representative of three independent assays. The hybridization and detection were conducted as described above.

### Nucleic acid binding assays.

RNA-binding studies were performed by EMSA. Five nanograms each of plus-strand AMV RNA3 5′-untranslated region (UTR) transcripts (5′UTR-WT BMV, 5′UTRΔ140, 5′UTRΔ105+5A, and 5′UTRΔ105+9A) was heated for 5 min at 65°C and cooled at room temperature for 10 min. Different amounts of purified six-histidine tag AMV CP (37) were added and incubated for 30 min at room temperature in a 10-μL final volume of PE buffer (10 mM Na-phosphate buffer [pH 7.0] and 1 mM EDTA) plus 5 U of human placental RNase inhibitor (63). Following incubations, 2 μL of tracking dye was added, and the samples were electrophoresed through 1.2% agarose gel at 50 V in TAE buffer (40 mM Tris-acetate, 1 mM EDTA [pH 8.0]). RNAs were transferred to positively charged nylon membranes (Roche Diagnostics GmbH, Mannheim, Germany) by capillarity in 10× SSC (1× SSC is 0.15 M NaCl plus 0.015 M sodium citrate) buffer overnight. RNAs were fixed to the membranes by UV using a cross-linker. Hybridization and detection of the digoxigenin (DIG)-RNA probe were conducted as previously described (62) with CSPD-Star substrate (Roche Diagnostics GmbH, Mannheim, Germany) using a riboprobe complementary to the 5′-UTR wild type. The ImageGauge 4.0 analyzer program was used to quantify the hybridization signals on the images.

### Western blot assay.

Approximately 2 × 10^6^ P12 protoplasts were centrifuged and resuspended with 125 μL of 1× Laemmli loading buffer (64). After boiling for 5 min, 25 μL of the mixture was subjected to 12% SDS-PAGE. Proteins were detected on Western blots using a polyclonal anti-AMV CP or anti-AMV MP from rabbit and a secondary anti-rabbit peroxidase-labeled antibody (Sigma) together with a chemiluminescence substrate (Amersham ECL Prime Western blotting detection reagent). The membranes were exposed to Kodak X-Omat AR film for ~30 to 45 min. Alternatively, the chemiluminescent signal was detected using the LAS 3000 imaging system from Fuji.

### Statistical analyses.

Prior to the pairwise *t* test comparisons, the homoscedasticity of variances was tested using a Levene’s *F* test. Depending on the result of this test, the corresponding *t* statistic and degrees of freedom were computed.
