# Supplementary material for: An Evolved 5′ Untranslated Region of Alfalfa Mosaic Virus Allows the RNA Transport of Movement-Defective Variants
Source: J Virol. 2022 Oct 31;96(22):e00988-22. doi: 10.1128/jvi.00988-22 (PMC9683001; doi:10.1128/jvi.00988-22)
Supplement: Supplemental file 1 — Fig. S1 and S2. Download jvi.00988-22-s0001.pdf, PDF file, 0.5 MB [file jvi.00988-22-s0001.pdf]

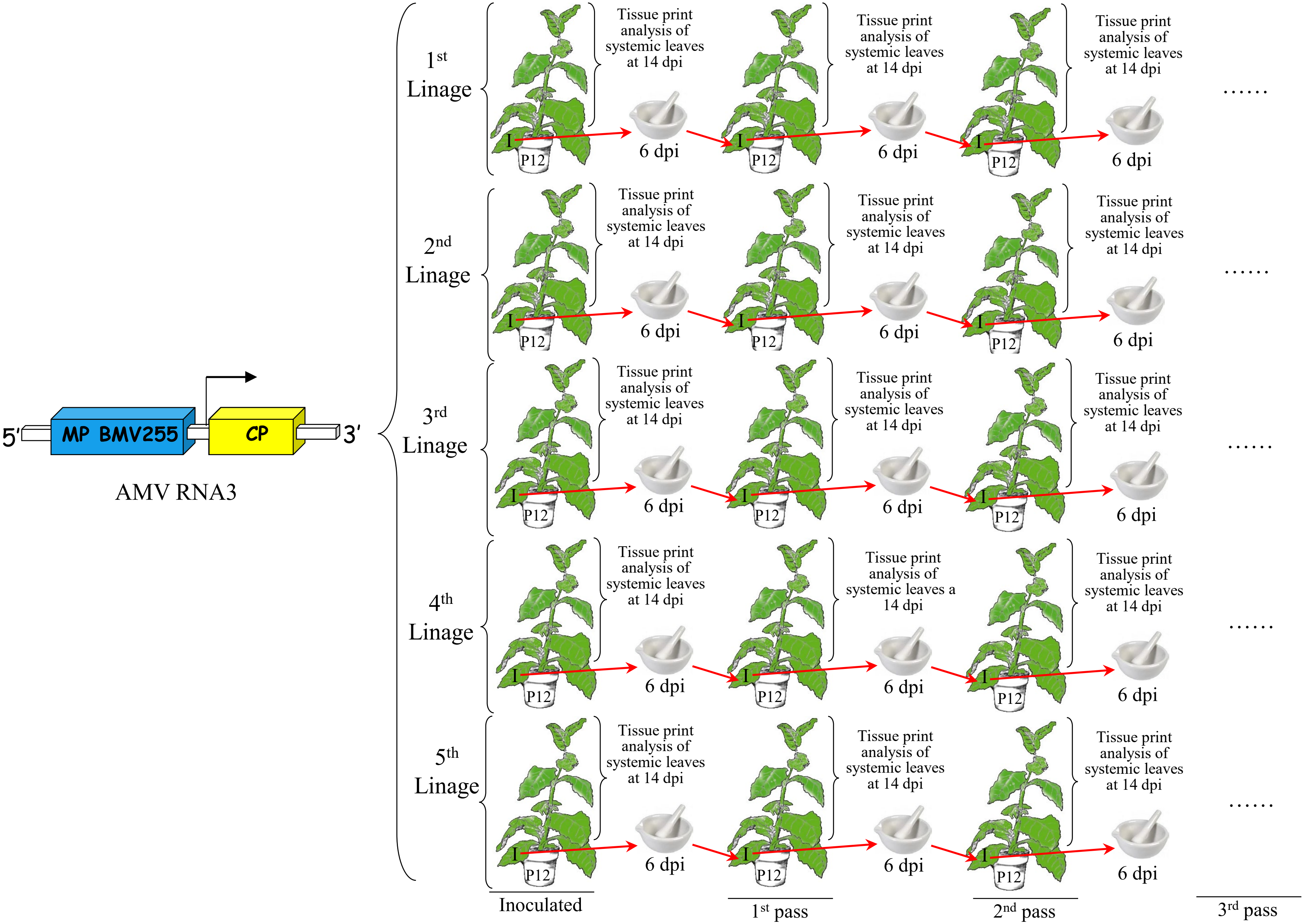

Figure S1. Schematic representation of the evolution experiment performed with the AMV RNA3 derivative unable to infect systemically the P12 plants. The scheme represents the AMV RNA 3 in which the MP of BMV, lacking the C-terminal 48 amino acids (MP BMV255), and the AMV coat protein (CP) are represented by blue and yellow big boxes, respectively. The small white boxes represent the 5'UTR and the 3'UTR whereas the subgenomic promoter is indicated by the arrow. Transcripts derived from the AMV RNA 3 were inoculated in 5 P12 plants, representing the first inoculated plant of the 5 independent lineages. At 6 dpi, the inoculated leaf (I) was used to inoculate the next P12 plant and at 14 dpi all systemic leaves were analyzed by tissue printing using a specific AMV probe complementary to the 3'UTR.

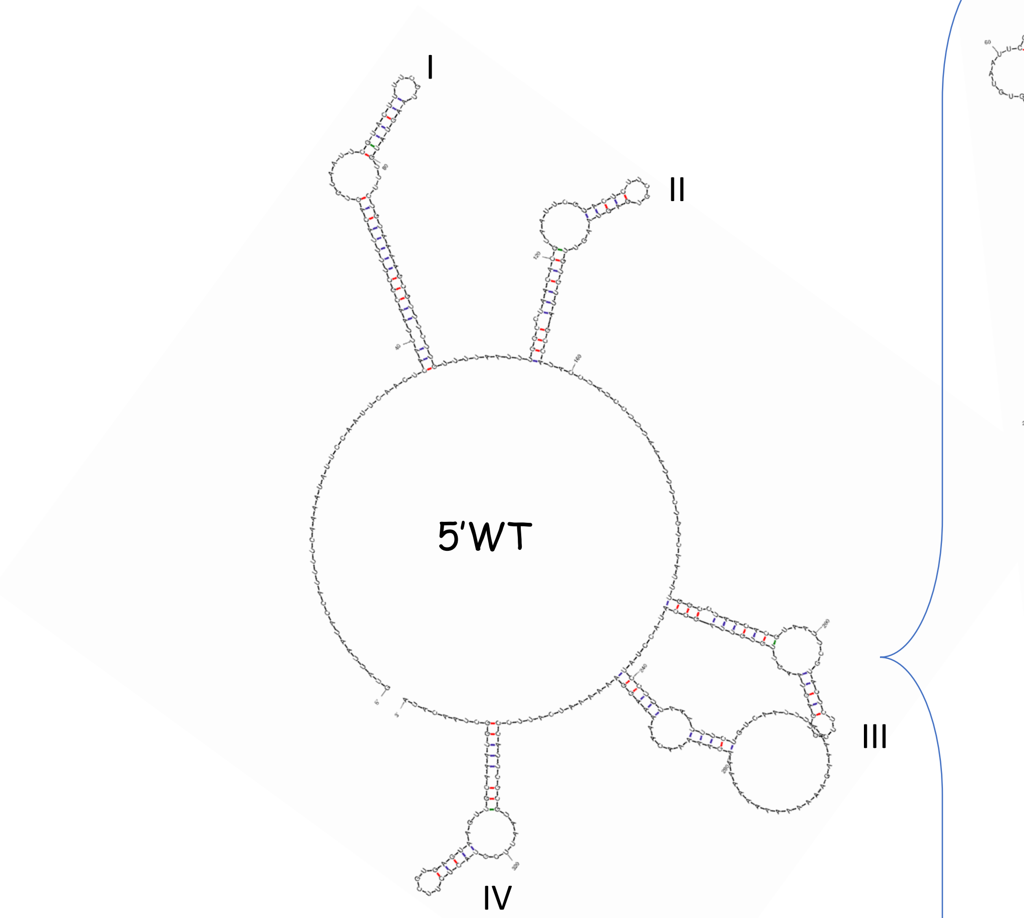

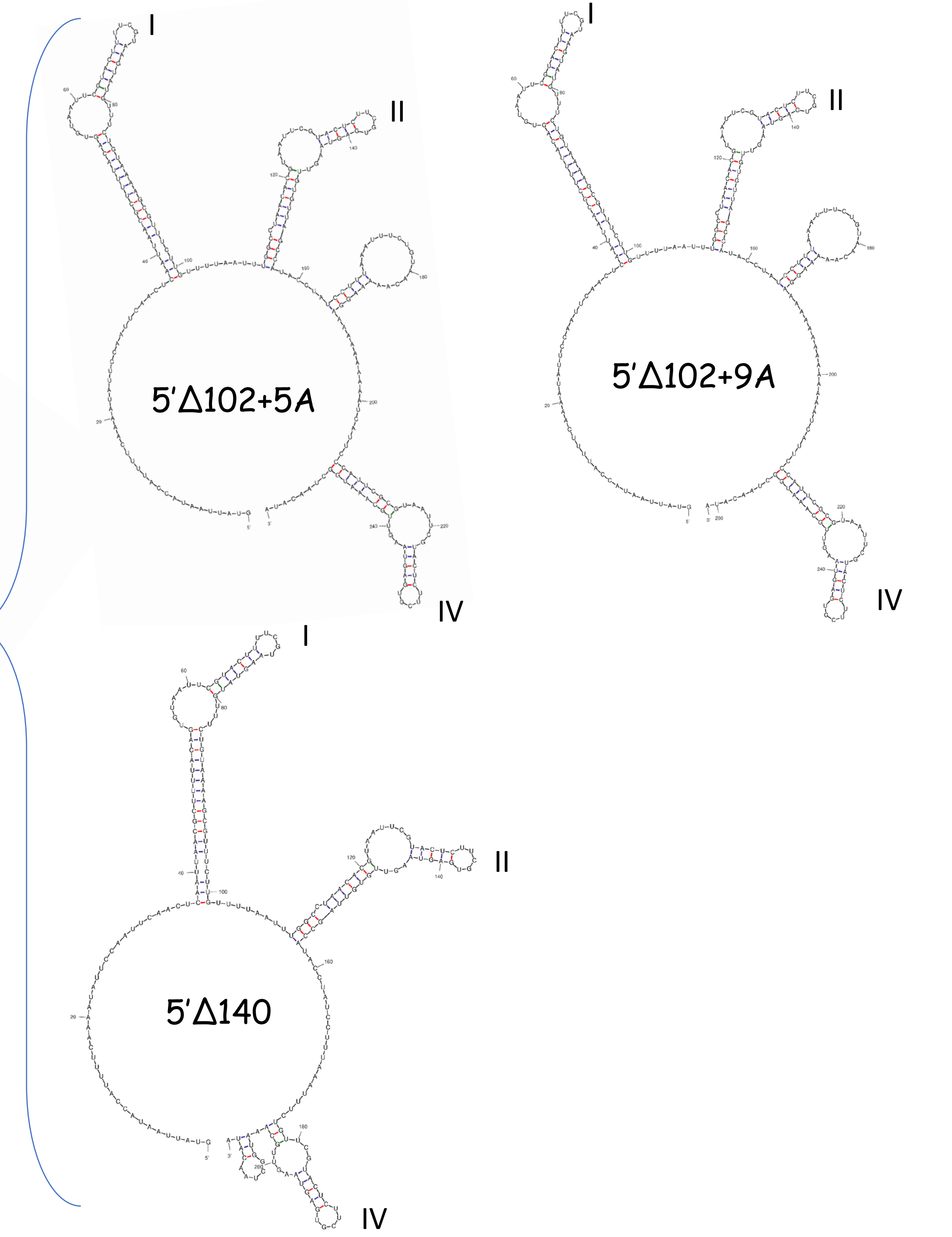

Figure S2. Minimal energy free structure of the 5'UTR of AMV RNA3 derivatives expressing the BMV MP and carrying the 5'UTR wild type (5'WT) or the 5'UTR mutants 5'Δ140, 5'Δ102+5A and 5'Δ102+9A. The analyzed 5'UTR encompass the full 5'UTR plus 9 nt downstream of the BMV MP start codon. The structure of the 5'UTR was generated using the Mfold software (64) (http://unafold.rna.albany.edu/?q=mfold/RNA-Folding-Form) using the default settings. The minimal energy structure of the 5'UTR wild type (5'WT) contains four stem loops (I, II, III, and IV) sharing identical nucleotides at the loop and part of the stem. An additional stem carrying a poly(A) loop was observed between stems III and IV. The stem loops conserved in the evolved 5'UTRs are indicated.
